# Childhood lead exposure in France: benefit estimation and partial cost-benefit analysis of lead hazard control

**DOI:** 10.1186/1476-069X-10-44

**Published:** 2011-05-20

**Authors:** Céline Pichery, Martine Bellanger, Denis Zmirou-Navier, Philippe Glorennec, Philippe Hartemann, Philippe Grandjean

**Affiliations:** 1 EHESP School of Public Health, CS 74312 - 35043 Rennes Cedex, France; 2 INSERM U 954 "Nutrition, genetics and environmental risks", Medical School, 9 av de la Forêt de Haye - BP 18 54505 Vandoeuvre-les-Nancy Cedex, France; 3 Nancy University Medical School, Public Health department, Vandoeuvre-les-Nancy Cedex, France; 4 Institute of Public Health University of Southern Denmark, J. B Winsloewsvej 17, DK-5000 Odense, Denmark; 5 Department of Environmental Health, Harvard School of Public Health, Boston MA 02215, USA; 6 IRSET-Research Institute for Environmental and Occupational Health-INSERM U625, Rennes, France

## Abstract

**Background:**

Lead exposure remains a public health concern due to its serious adverse effects, such as cognitive and behavioral impairment: children younger than six years of age being the most vulnerable population. In Europe, the lead-related economic impacts have not been examined in detail. We estimate the annual costs in France due to childhood exposure and, through a cost benefit analysis (CBA), aim to assess the expected social and economic benefits of exposure abatement.

**Methods:**

Monetary benefits were assessed in terms of avoided national costs. We used results from a 2008 survey on blood-lead (B-Pb) concentrations in French children aged one to six years old. Given the absence of a threshold concentration being established, we performed a sensitivity analysis assuming different hypothetical threshold values for toxicity above 15 μg/L, 24 μg/L and 100 μg/L. Adverse health outcomes of lead exposure were translated into social burden and economic costs based on literature data from literature. Direct health benefits, social benefits and intangible avoided costs were included. Costs of pollutant exposure control were partially estimated in regard to homes lead-based paint decontamination, investments aiming at reducing industrial lead emissions and removal of all lead drinking water pipes.

**Results:**

The following overall annual benefits for the three hypothetical thresholds values in 2008 are: €22.72 billion, €10.72 billion and €0.44 billion, respectively. Costs from abatement ranged from €0.9 billion to 2.95 billion/year. Finally, from a partial CBA of lead control in soils and dust the estimates of total net benefits were € 3.78 billion, € 1.88 billion and €0.25 billion respectively for the three hypothesized B-Pb effect values.

**Conclusions:**

Prevention of childhood lead exposure has a high social benefit, due to reduction of B-Pb concentrations to levels below 15 μg/L or 24 μg/L, respectively. Reducing only exposures above 100 μg/L B-Pb has little economic impact due to the small number of children who now exhibit such high exposure levels. Prudent public policies would help avoiding future medical interventions, limit the need for special education and increase future productivity, and hence lifetime income for children exposed to lead.

## Background

Lead is a well known toxic metal, and current exposures in children constitute a reason for concern [1]. In France, lead has multiple anthropogenic sources and is now mainly present in its inorganic form in the environment [2,3]. The relative importance of different sources depends on the blood lead range. For the general European population [1] and for children [4], food is usually the major source of exposure, with cereals and vegetables products contributing mostly to dietary lead exposure. Tap water can also, in some cases, be an important contributor because of the presence of lead pipes in old homes and public plumbing systems. Degradation of old lead-based paint results in the contamination of indoor dust that can be inhaled or ingested, thus adding to the sources already mentioned. Other incidental sources of lead exposure include consumer products, notably toys, and hobbies or occupations involving lead [3]. After the ban of leaded petrol, air concentrations have decreased substantially and are now due almost entirely to industrial emissions [5,6]. In France, the targeted regulations to decrease elevated B-Pb concentrations, control measures and screening strategies have progressively reduced risks from lead pipes, lead-based paint in houses built before 1949 and contamination at specific industrial sites [3]. Children under six years of age have the highest exposure to lead because of several factors such as greater hand dust contamination, frequent hand-to-mouth transfer and higher absorption rates than adults. Also, lead can pass through the placenta so that the child is born with lead from the mother's cumulated body burden [7]. Overall, lead poisoning is still a serious hazard for children and causes significant neurologic damage linked to cognitive and behavioral impairment [1,8]. Although frequently overlooked, the timing of the dose in regard to windows of highest vulnerability in children is also important [9,10].

The first national study carried out in France in 1999 by the National Institute of Health and Medical Research (INSERM) showed that 2% of French children aged one to six years of age had B-Pb concentrations > 100 μg/L (i.e. approximately 85,000 children); the geometric mean blood-lead concentration was 37 μg/L [2]. This exposure level was similar to other Western European countries [11]. In a new survey, 2008-2009, the National Institute for Health Surveillance (InVS) [12] found that the geometric mean B-Pb had decreased to 15 μg/L (standard deviation [SD], 1.6) among children aged 1-6 years, and the prevalence of B-Pb concentrations > 100 μg/L had dwindled to 0.11% (i.e. 5,333 children) [12]. Nonetheless, many children are still at risk because there is no evidence for a lead toxicity threshold. The B-Pb concentration intervention value in the US and France is 100 μg/L; above this limit the subject is considered as lead poisoning by public health authorities and is supposed to be reported in the French National system of surveillance of children's B-Pb concentrations. At lower values lead toxicity may still cause damage to nervous system functions, including decreased nerve conduction velocity and cognitive deficits [1], and significant neurologic damage may occur as a result of both intrauterine and postnatal exposures [13,14]. The intellectual decrement may be expressed in terms of a loss of IQ points for every μg/L unit increase of the B-Pb, but this loss slope is steeper at B-Pb concentrations lower than 100 μg/L than at higher levels [14]. At the individual level, this drop may seem small and inconsequential, but at the population level, small effects in many individuals are likely to have an impact on the overall societal benefits [11]. The effects include lower school performance and educational attainment, which may influence societal adaptation and economic success, with some affected children showing juvenile delinquency [11,15]. Therefore, improvements in cognitive ability will benefit society by raising both economic wealth and overall wellbeing. Several economic studies, mainly in the US, have estimated the costs and risks associated with infantile lead poisoning and lead toxicity, in some cases weighing them against the costs associated with lead-based paint control and other efforts. These studies have also calculated the potential increased financial earnings that would result if the level of lead in children's blood were to be reduced [[8,16], and [17]]. In France, studies are mostly epidemiological, focusing on targeted screening and lead exposure. There have been few economic assessments of lead's impact on the children's health, with the exception of the studies by Chanel [18-20], while Fassin and colleagues highlighted the social aspects of lead exposure [21]. The present paper aims to fill the gap and contribute at least in part to a cost benefit analysis (CBA), while taking into account that there is "no single estimate that accurately reflects the costs and the benefits of lead hazard control" [8]. We first summarize the childhood lead exposure situation in France and related information on the main exposure media and risk factors. We then estimate the monetary benefits that can be expected from pollutant abatement, with estimates of investment costs to achieve this reduction, as based on available information. Lastly, we compare the main findings of this study and discuss the role of CBA in a societal perspective of public policy development.

## Methods

### Population studied and sources of lead exposure

We based our estimations on the InVS study [12]. The geometric mean of children's B-Pb concentrations in France was found to be 15.1 μg/L, with a SD of 1.6 (log-normal distribution). We used the same target population consisting of 4.7 million children from one to six years of age according to the National Institute for Statistics and Studies [12]. Table 1 shows the distribution and the number of children exceeding the hypothetical threshold values for this cohort. Estimates were made based on the entire cohort in order to highlight the global economic impact on the most sensitive segment of the population to lead exposure. Derived from this estimate, the size of the population experiencing lead poisoning (at B-Pb ≥100 μg/L) was 5,333 [12]. We used data from the French National system of surveillance of children's B-Pb concentrations (SNSPE, 2005-2007) [22] to assess the distribution of risk factors among children with B-Pb concentrations ≥ 100 μg/L. Based on the SNSPE data, 74% of the cases were associated with poor housing: old buildings (i.e. those built before 1949), degraded, with humidity and lead-based paint still present on walls or windows and door frames. Another 4% were estimated to be linked to industrial emissions and only 1% to contaminated water. However, it is worth noting that these data rely upon screening programmes whose results may vary according to the main sources of exposure in different regions, and also according to the screening strategy. For example, in the Paris region, the main exposure media for high (≥100 μg/L) B-Pb concentrations were contaminated dust and soils. In comparison, exposure of the screened children in the North of France region was mainly linked to the old Metaleurop smelter which represented 42% of all screened cases. Based on these same data, all regions included, we thus considered that contaminated soils and dust or ingested flakes from degraded paint in old homes <1949 were the main risk factor in three out of four cases for B-Pb concentrations ≥ 100 μg/L. These results are in line with US data where 70% of cases with high B-Pb concentrations were due to lead-based paint [23].

**Table 1:** Estimates of total direct health costs within B-Pb concentration ranges for the French child population (€_2008_)

Blood-lead concentrations range (μg/L)	**% of children aged 1 to 6 years**^**a**^	**Number of children**^**a**^	Unit cost (€)	Total costs (€ million)
B-Pb < 15	50.00	2,348,091	0	0

15 ≤ B-Pb < 24	35.1	1,648,975	120	198

24 ≤ B-Pb < 100	14.8	693,783	120	83

B-Pb ≥ 100	0.1	5,333	2,932	16

^a ^On the basis of INSEE data and INVS results, 2010

Table 1 shows the direct health cost B_med _within B-Pb concentration ranges for the French child population. B_screening 15-24 _and B_screening24-100 _amount to 120 € per child and B_treatment≥ 100 _is estimated to €2,932 which equals to ((1,819*0.73+4,851*0.27) +294) per child.

Now, 99% of children from one to six years old have B-Pb concentrations <100 μg/L (Table 1). Glorennec and colleagues [4] estimated the fractions of exposure due to different sources for this population under ordinary exposure conditions. We selected these data to assess the contribution of the most prominent risk factors at the 75^th ^percentile of the distribution (P75). Food was found to constitute the main exposure medium (83%), followed by dust and soil (16%) and water (1%).

### Assessment of IQ decrements

Environmental lead exposure in children may cause cognitive impairment among children ≤ 6 years, as assessed by measurement of IQ. The international pooled analysis by Lanphear and colleagues [14] established a non-linear, negative relationship between IQ and B-Pb concentrations. Between 24 and 100 μg/L, the decrement per unit of μg/L increase in B-Pb amounted to 3.9 IQ points (95% CI, 2.4-5.3). At higher exposures, i.e. from to 100 to 200 μg/L, and from 200 to 300 μg/L, the drop in IQ points was 1.9 (95% CI, 1.2-2.6), and 1.1 (95% CI, 0.7-1.5), respectively. Thus far, there are few studies so far examining exposures below 24 μg/L. However, as concluded by the European Food Authority Safety (EFSA): "no threshold for these effects has been identified, and the evidence suggests that the response at B-Pb concentrations below 100 μg/L is steeper than at higher exposure levels" [1]. In addition, a recent risk assessment study by the California Environmental Protection Agency (CEPA) calculated that a 10-μg/L increase in B-Pb in the range of 10-100 μg/L resulted in a population-level decrement of one IQ point [24,25].

Given that no threshold for lead toxicity has been established, we conducted a sensitivity analysis assuming that loss of IQ in the study population starts at values exceeding 15 μg/L, respectively 24 and 100, following a "what if ?" approach; the first value is close to the geometric mean of B-Pb among French children (15.1 μg/L) [12]. We assumed a loss of one IQ point from 15 to 24 μg/L. And further used the dose-effect decrements calculated by Lanphear and colleagues for values from 24 to 100 μg/L, and a loss of 1.9 IQ points from 100 μg/L to 200 ug/L.

### Cost Benefit Analysis

Cost benefit analysis (CBA) is often used in health care assessment, as it links the costs of a strategy to its results or benefits expressed in monetary units. The rationale of CBA implies that an intervention should be undertaken if the sum of its benefits (B) is greater than the sum of its costs (C). An alternative way of expressing this is to say that its net benefit (B-C) is positive or that its B/C ratio is greater than 1. The preferred option will be the one which maximizes this net benefit, and consequently the new CBA-based health strategy will provide a net benefit to society [26-28].

**Figure 1:**
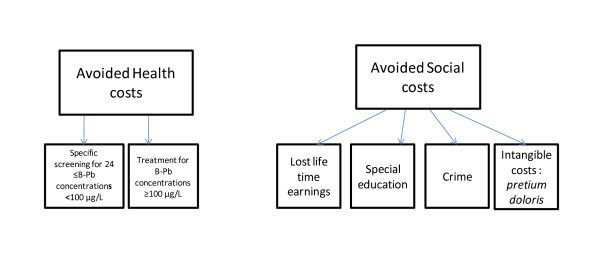
**Monetary benefits assessed in terms of avoided costs**.

For this study, we based our estimation on the yearly economic impact of reduction of lead exposure for each birth cohort (children born within one calendar year) and compared these social benefits to investments needed to reduce exposure and control risk factors. Because little information is available on the investments required in France to abate lead exposure, we focused our evaluation on the benefit side, and provided preliminary estimates of costs of exposure abatement. We assessed the benefits in terms of avoided costs (see Figure 1).

The calculation of benefits took into account the range of B-Pb concentrations above the thresholds already defined. These estimates of benefits B are defined as follows:(1)

where B_med _are the direct avoided costs; B_earn_/w are the lost lifetime earnings, applying a discount factor w equal to (1+s) ^t^, with a 3% discount rate (s) to a time horizon t about 30 years; B_spec.ed _are the costs for special education; B_crime _are the costs due to juvenile delinquency - the latter three being social benefits; and B_other _are the intangible costs. For our estimations, we used the purchasing parity power (GDPppp$-€) when applying American cost data in the French setting. The estimates are inflation adjusted [26,29,30] and performed for one year (2008).

### Annual benefit estimation

#### Health benefits: costs of avoiding lead poisoning

Based on the InVS data B-Pb distribution (Table 1), we estimated direct costs B_med _from the component costs B_screening15-24_, B_screening24-100 _and B_treatment ≥ 100 _for screening and treatment within the observed B-Pb range (15-24 μg/L as "15-24", 24-100 μg/L as "24-100" and ≥ 100 μg/L as "≥ 100", respectively). We calculated B_screening15-24_, B_screening24-100 _and B_treatment ≥ 100 _as costs of screening, hospitalization, and medical consultations within the observed B-Pb range and in agreement with the French recommendations [31] for children aged six months to six years. Part of these costs were based on a pilot study undertaken by the Aubervilliers local authority, which provided reference costs for 2006, that were inflation-adjusted for 2008 [3]. B-Pb < 15 μg/L was considered as not requiring medical attention. Since treatment is used only for children above 100 μg/L, subjects with lower values incur only screening costs which amount to 120 € per child in 2008, labeled as B_screening 15-24 _and B_screening24-100_, respectively. The direct health cost estimates for B-Pb ≥ 100 μg/L up to 300 μg/L is given by B_treatment ≥ 100_. In this case, the screening cost per child was estimated from €1,819 for screened children (73% of all cases) to €4,851 for new cases of lead toxicity (27% of all cases [3]). We also added unit costs for medical follow-up: €294, medication included, according to Brown [32].

Unit cost estimate of outpatient chelation treatment, without medication, was €3,491 of which €2,365 and €1,126 for nursing follow-up and in-home hospitalization, respectively. This cost should be added to screening costs for children with B-Pb concentrations ≥300 μg/L [33]. Hence, B_treatment ≥ 100_, equal to €2,932 [(1,819*0.73+4,851*0.27) +294] for lead toxicity at B-Pb ≥ 100 μg/L, should be €6,423 (2,932+3,491) for B-Pb ≥ 300 μg/L. However, due to the lack of information on the number of children with B-Pb concentrations ≥300 μg/L in the InVS study, we assumed that all screening costs were €2,932 for B-Pb ≥100 μg/L.

#### Indirect economic benefits related to health improvement

In our case, part of the indirect costs represents the productivity losses to society due to lead toxicity. For the purpose of this study, the indirect costs include the loss of lifetime earnings, the costs of special education, and the costs of juvenile delinquency.

#### IQ and lost lifetime earnings due to lead poisoning

The lifetime costs associated with lower earning potential caused by lead toxicity is based on a linear relationship between the loss of IQ due to lead and expected lifetime earnings. From the studies by Lanphear and colleagues [14], and the CEPA study [24], we assumed 1 IQ point loss from 15 to 24 μg/L. According to Lanphear's IQ decrements, we used 3.9 IQ points from 24 to 100 μg/L, to which, we added the first IQ point loss, (1+3.9 = 4.9). We applied an average IQ point loss of 3.9/7.6 = 0.51 point per 10 μg/L within this range. According to the available data in [12], we used percentile values for the intermediate calculations between 24 and 100 μg/L. Above 100 μg/L, the IQ point loss was 6.8 (= 1+3.9 +1.9) per 100 μg/L (Table 2). Although the IQ and the B-Pb assessments were usually made at 7 years, similar associations were found for lead concentrations at younger ages, but they are considered less stable [14,34]. We therefore applied these IQ losses to the selected 1-6 years children. We followed Gould's method where estimates of IQ decrement were based on the data from the main published studies [16,17,35], and we drew from her 2006 estimate of $_2006 _17,815 for the present value of incremental lifetime earnings. We adjusted it for inflation to €_2008 _and the loss was thus estimated to be €17,363 per IQ point in 2008. Again,(3)

**Table 2:** Lifetime earning losses per year of the selected cohort according to IQ point losses within B-Pb concentration ranges (€_2008_)

Blood-lead concentrations range (μg/L)	**IQ point loss assumptions **^**a, b, c**^	**Number of children **^**d**^	Number of IQ point losses	**Total Costs (€billion)**^**e**^	**Lost life time earnings with a discount factor w**_**30**_**(€billion)**
B-Pb < 15	0	2,348,091	0	0	0

15 ≤ B-Pb < 24	1	1,648,975	1,648,975	28.6	11.8

24 ≤ B-Pb < 100	4.9 (1+3.9)	693,783	1,421,769	24.7	10.2

B-Pb ≥ 100	6.8 (1+3.9+1.9)	5,333	36,265	0.6	0.3

TOTAL		4,696,182	3,107,009	53.9	22.3

Based on ^a ^EFSA conclusions [1], ^b ^CEPA [24], ^c ^Lanphear and colleagues. [14], ^d ^InVS data [11] and ^e ^Gould [8]

Table 2 presents lifetime earning losses per year of the selected cohort according to IQ point losses within B-Pb concentration ranges. The IQ point loss assumptions were 1, 4.9 (= 1+3.9) with 0.51 point per 10 μg/L within this range, and 6.8 (= 1+3.9 +1.9) IQ point losses per 100 μg/L within this range, from 15 to 24 μg/L, from 24 to 100 μg/L, and above 100 μg/L respectively. The loss per IQ point was estimated to be €_2008_17, 363. Based on the equation 3 (B_earn _= B_earn15-24 _+ B_earn24-100 _+ B_earn≥100_), the total lost lifetime earnings due to lead toxicity B_earn _were estimated, with B_earn15-24 _for increased B-Pb <24 μg/L, B_earn24-100 _for B-Pb between 24 μg/L and 100 μg/L, and B_earn≥100 _for B-Pb ≥100 μg/L. We applied a discount factor w_30 _on the total costs and we obtained € 22.3 billion, € 10.5 billion and € 0.3 billion, respectively for the year 2008.

where B_earn _are the total lost lifetime earnings due to lead toxicity, with B_earn15-24 _for increased B-Pb < 24 μg/L, B_earn24-100 _for B-Pb from 24 μg/L to 100 μg/L, and B B_earn≥ 100 _for B-Pb ≥100 μg/L.

#### Special education

Children with elevated B-Pb concentrations have an increased risk of enrollment in special education. Two categories of French institutions take care of children and young adults between three and 20 years old with cognitive and behavioral impairment. The Medical Educational Institutes (IME) educates children with intellectual deficiency symptoms while the Educational and Therapeutic Institutes (ITEP) do so for behavioral problems. According to Schwartz [16], 20% of children with B-Pb > 250 μg/L need special education. A more recent study suggested that the need for such service could start below this concentration [36], i.e. when B-Pb exceeds 100 μg/L. Lyngbye and al. showed that, even at low levels of lead exposure, the need for special education increases with the exposure level [37]. Another reference also showed for children with B-Pb≥100 μg/L lower intelligence and behavior changes [38]. From their findings, we estimated the need for special education to be 10% for children with B-Pb ≥ 100 μg/L, the cost denoted B_spec.ed≥ 100_.

The French national data show that 79.8% and 20.1% children with cognitive and behavioral deficiencies are in IME and ITEP, respectively [39]. The estimated average annual cost per child was €38,958 in IME and €48,255 in ITEP in 2008 [40].

#### Violent behavior leading to juvenile delinquency

The Nevin's study [36] recent evidence of a link between prenatal and early-childhood lead exposure and increased risk of criminal behavior later in life illustrated that showed a strong association between preschool B-Pb and subsequent crime rate trends over several decades in various countries, including France. The relationship was characterized by best-fit lags consistent with neurobehavioral damage in the first year of life and the know peak age of offending for index crime, burglary, and violent crime [36,41]. Several other studies support the link between preschool lead exposure and aggressive or delinquent adolescent behavior and subsequent criminal violence [42,43]. We therefore estimated the costs linked to lead-associated crime on the basis of Gould's approach [8]. We first obtained the total number of violent/aggressive specific crimes committed in 2008 from the French national observatory of Delinquency [44]. We then used data from Nevin [36] to estimate the share of each of the crimes that might be associated with lead toxicity. These include burglaries (2.4%), robberies (0.7%), aggravated assaults (3.1%), rape (2.7%), and murder (5.4%). The total lead-linked crimes were computed on the basis of the French population aged 13-60 years liable to commit a violent act [45]. We next calculated (B_crime_) the costs directly associated with each sort of crime and the total cost of lead-linked crimes.

We used French data when available, and otherwise US data in the absence of French data for direct costs of victims and overhead costs of justice and incarceration and for lost earnings for both criminals and victims, as well [46,47]. All costs were adjusted by the ratio of US and French crime rates (the US rate crime of 5.6 per 100,000 being much greater than the French rate crime, 1.7 per 100,000, in 2005) [48,49]. In this case,(5)

where B_crime _are the cost estimates for B-Pb ≥100 μg/L

#### Intangible costs

In addition, suffering and degradation of the quality of life associated with lead poisoning and its side effects had to be taken into account. Intangible costs, mentioned B_other_, while difficult to measure were taken into account using the "pretium doloris" approach. These costs were estimated for children with B-Pb concentrations≥ 100 μg/L. The Metal Blanc factory of Bourg-Fidèle (Ardennes administrative subdivision, North East France), specializing in the recovery of lead from used batteries (drum kits), was condemned for putting lives at risk in September 2009. The judge called for €120,000 of 'damages and interests' to the victims, the cases of six families having been declared valid. The judgment called for €8,000 for each child with B-Pb concentrations≥ 100 μg/L [50]. We note:(6)

#### Annual total benefits

In summary, the total benefits (avoided costs) are therefore given by(7)

Final estimation included confidence intervals and a sensitivity analysis using different key assumptions from the American and European data, on which the calculations were based. Benefits were estimated according to different B-Pb hypothetical threshold values, i.e. 15 μg/L, 24 μg/L and 100 μg/L, respectively.

### Abatement cost estimation

#### Estimates of costs associated with reduction of B-Pb concentrations

Due to insufficient cost data related to control of lead hazards, only preliminary estimates of cost incurred by pollution control were performed, as indicated below. We estimated total lead-based paint decontamination costs, partial costs of industrial emission abatement and lead pipe removal costs.

#### Total lead-based paint decontamination costs to remediate French houses

These costs, denoted C_paint_, were calculated on the basis of InVS [3] and INSEE data [2] on 37,382 lead-paint based homes and using an average estimated removal cost per home. According to the SNSPE data [22] and to Glorennec and colleagues, [4] lead in soils and dust from the lead-based paint in homes built before 1949 represent 74% and 16% of cases of childhood lead intoxication for blood lead levels greater and lower than 100 μg/L, respectively. We estimated the costs of decontaminating French houses with lead-based paint following the data from the national Agency of the housing environment (ANAH) scenarios regarding elimination of lead presence. Only 37,382 homes had to be decontaminated among about 28 million French homes: therefore we considered that these operations could be performed once and for-all in one year's time.

#### Industrial investments costs to reduce lead exposure

The costs of investments (denoted C_ind_) to control industrial lead pollution and reduce lead emissions both in air and water were also estimated. They include technologies to recycle and reduce presence of lead in batteries and in glass, abatement of diffuse emissions through increase in the efficiency of recycling, capture and treatment of the contaminated discharges. Investment costs were weighted per factory volumes based on data from a National Institute for Industrial Environment and Risks - (INERIS) [51]. These were annual costs.

#### Costs to eliminate water lead pipes

These costs, denoted C_water_, were estimated following the High Council of Hygiene (CSHPF) and the French Food Safety Agency (AFSSA) recommendations for removing all lead pipes used in public water supply and in household plumbing, in order to reach a lead concentration of ≤10 μg/L before the end of year 2013. C_water _based on the estimations of the European Institute Reasoned Management for the Environment (IEGRE) [52], C_water _was found to be €10 billion for household pipes, and €4 billion for public pipes. We calculated an investment plan over five years to reach the above mentioned objective, (denoted C_pwater_). Although a longer investment plan could have been chosen, we calculated the annual costs for an investment plan over 5 years to cover the expenses. We used ANAH estimates and French or US data, according to which were available [3,53-55].

## Results

### Annual Benefits

Direct health care costs were estimated in accordance with equation (2) and were found to be €0.297 billion/year as shown in table 1. Direct health costs represented 0.14% of the total French health expenditure in 2008. Table 1 reports the direct health cost estimates B_screening15-24_, B_screening24-100 _and B_treatment≥ 100 _per B-Pb concentrations range.

Lost lifetime earnings ranged from €0.6 billion (B_earn≥ 100_) to €53.3 billion (B_earn15-24 + _B_earn24-100 _) according to B-Pb concentrations ≥ and <100 μg/L, respectively, as presented in Table 2. Thus, B_2 _estimates were € 53.9 billion per year for the full B-Pb range. We note that the loss of IQ associated with B-Pb concentrations between 15 μg/L and 100 μg/L amounted to more than 99% of the total estimated costs. Thus, the loss of IQ would be marginally influenced by the number underestimating of children having a high B-Pb ≥100 μg/L. Applying the discount factor w (w_30_= (1/(1.03)^30^)) on lost life-time earnings, we obtained the estimate: € 22.3 billion above 15 μg/L, € 10.5 billion above 24 μg/L and € 0.3 billion above 100 μg/L.

For special education, the annual national cost estimate B_spec.ed≥ 100 _was € 14.53 million for 10% of children with B-Pb concentrations ≥100 μg/L in need of special education.

For deviant behavior and crime, a reduction of 10 μg/L in preschool B-Pb ≥100 μg/L would result in 4,770 fewer burglaries, 102 fewer robberies, and 2,206 for aggravated assaults, 171 for rapes, and 29 for murders. In France, the total estimated cost of lead-linked crimes (B_crime≥ 100_) was approximately €61.8 million per year, as shown in Table 3, this accounted for 0.3% of the total cost of crime in 2008 [46].

**Table 3:** The effect of developmental lead exposure on crime in France and the associated annual costs (€_2008_)

Crime	**Number of crimes per 100,000 French residents (N) **^**a**^	**Lead linked crimes per 100,000 French residents (N) **^**b**^	Total lead linked crimes (N)	**Costs per crime (e) **^**c, d**^	Total direct costs€million
Burglaries	497.9	11.7	4,770	2,004	9.6

Robberies	37.79	0.3	102	22,529	2.3

Aggravated assaults	172.8	5.4	2,206	20,058	44.3

Rape	15.5	0.4	171	27,990	4.8

Murder	1.33	0.1	29	30,645	0.9

a: calculated using data from the National Observatory of the delinquency, 2009[44] b: (Nevin, 2006) by using French rate crime[36] c: calculated data from (Arlaud, 2006)[46] d: calculated data from the US Bureau of Justice Statistics inflated to 2008[47].

Table 3 shows the effect of developmental lead exposure on crime in France and the associated annual costs. We first informed on the number of the selected crimes per 100,000 French residents committed in 2008: 497.9 burglaries, 37.79 robberies, 172.8 aggravated assaults, 15.5 rapes and 1.33 murders. US Lead linked crimes (with US crime rate (5.6 per 100,000)), estimated by Nevin, were adapted to the French crime rate (1.7 per 100,000): we obtained 11.7(e.g. =(38.7/5.6)*1.7) burglaries, 0.3 robberies, 5.4 aggravated assaults, 0.4 rape and 0.1 murder for lead linked crimes per 100,000 French residents. We calculated the total lead linked crimes for the French population aged 13-60 years. We then used French and US available data for the direct costs per crime and multiply these latter with total lead linked crimes to obtain the total direct costs per year (€61.8 million in 2008).

Intangible costs for the population with B-Pb ≥100 μg/L were calculated as compensations, resulting in a total cost of €42.7 million (B_other≥ 100_).

Based on these estimates, we calculated the total benefit of prevented lead toxicity as the sum of avoided costs. They included specific screening and treatment costs of lead poisoned children (€0.3 billion), lost lifetime earnings (€53.9 billion), special education costs (€0.145 billion), intangible costs (€0.0427 billion), and the direct costs related to crime (€0.0618 billion). We obtained the following total benefits for the three sensitivity analyses hypothetical threshold values of 15, 24 and 100 μg/L: € 22.72 billion, € 10.72 billion and € 0.44 billion, respectively, in 2008 (Table 4). The social benefits represented 98.7%, 99% and 96.5%, respectively of the total benefits. A unit benefit was estimated per child and per different B-Pb concentration values, as follows €9,676, €15,334 and €82,505, respectively, for the three threshold assumptions.

**Table 4:** Total Benefits and total cumulated benefits per year (in €_2008 _Billion)

Blood-lead concentrations range (μg/L)	Bmed	Bsocietal	Total benefits	Hypothetical threshold values (μg/L)	Total cumulated benefits
15 ≤ B-Pb < 24	*0.198*	*11.8*	11.99 (1)	B-Pb ≥ 15	22.72 (1+2+3)

24 ≤ B-Pb < 100	*0.083*	*10.2*	10.28 (2)	B-Pb ≥ 24	10.72 (2+3)

B-Pb ≥ 100	*0.016*	*0.44*	0.44 (3)	B-Pb ≥ 100	0.44

Table 4 shows the estimated total benefits ranged from blood-lead concentrations and total cumulated benefits based on three hypothetical values per year. We first differenced the estimated medical benefits (Bmed) and the societal benefits (Bsocietal) ranged from blood-lead concentrations:

The 15-24 μg/L Bmed, the 24-100 μg/L Bmed and the ≥ 100 μg/L Bmed are the B_screening15-24 _(€0.198 Billion), the B_screening24-100 _(€0.083 billion) and the B_treatment≥ 100 _(€0.016 billion), respectively.

The 15-24 μg/L Bsocietal, the 24-100 μg/L Bsocietal and the ≥ 100 μg/L Bsocietal are the B_earn15-24 _discounted (€11.8 billions), _The _B_earn24-100 _discounted (€ 10.2 billions) and the B_earn≥ 100 _discounted added to the B_spec.ed≥ 100 _, the B_crime≥ 100 _and the B_other≥ 100 _(€0.44 billion), respectively. The B_spec.ed≥ 100 _equal to €0.01453 billion [(10% of the French population of children 3-6 years) ((79.8%*38,958) + (20.1%*48,255))], the B_crime≥ 100 _equal to € 0.0618 billion and the B_other≥ 100 _equal to €0.0427 billion, which are the intangible avoided costs. We estimated the total benefits (Bmed +Bsocietal) ranged from blood-lead concentrations: €11.99 billions (1), €10,28 billions (2) and € 0.44 billion (3).

We secondly estimated total cumulated benefits per year based on the three hypothetical threshold values, above 15, 24 and 100 μg/L. We obtained €22.72 billions (1+2+3), €10.72 billions (2+3) and € 0.44 billion (3), respectively.

### Abatement Costs

Table 5 shows that lead-based paint decontamination costs per home ranged from € 3,562 to €9,162, with €6,562 as the central estimates, giving total cost estimates C_paint _from €245.3 [€133.1; €342.5] million in 2008. The annual industrial costs estimated C_ind _would have been €28.9 million in 2008. For water lead pipes, the total estimated costs C_water _between €4 billion and €14 billion. We applied a 3% discounting rate for C*_paint_+C*_ind _and an investment plan P on five years for C_water_. Hence, on the basis on available data, annual estimates of total costs of lead hazard control C*_paint_+C*_ind _+C_P water _ranged from €0.9 billion to 2.95 € billion. Reported per child within the cohort a unit cost was estimated to range from €185 to €629.

**Table 5:** Costs to decontaminate French houses with lead-based paint (€_2008_)

Type of costs	Cost1 per home	Cost2 per home	Cost3 per home
Global environmental survey	381^a^	381^a^	381^a^

Home dust analysis	30^b^	30^b^	30^b^

Home paint analysis	30^b^	30^b^	30^b^

ANAH's assumptions	2,600^c1^	5,600^c2^	8,200^c3^

Housing substitutes	521^d^	521^d^	521^d^

Overall interventions	3,562	6,562	9,162

Total costs (€million)	133.1	245.3	342.5

a = Argeron, 1995, actualized in 2008 by INVS [3]. b = LERES, 2009[54]. c = The National Agency of the housing environment (ANAH)[53], 2010., d = Mc Laine and colleagues.,2006, €2008[55].

Table 5 presents lead-based paint decontamination costs per home. We used French data for global environmental survey (€381) and for home dust and home paint analysis (€30, each one). We used also the assumptions of ANAH works for estimating the removal of lead-based paint cost per home eliminating lead. These assumptions were the following ones: Assumption 1: a 20% max rate was applied to €13,000 standard works for rehabilitating old houses <1949, irrespective any lead-based paint intervention. Assumption 2: a 70% max rate was applied to €8,000 works of specific lead decontamination Assumption 3: Assumptions 1 & 2 combined, i.e. the max mix of two works.

The housing substitutes, € 521, were US data based on Mc Laine analysis. Based on these data and assumptions, we calculated three overall interventions ranged from €3,562 to €9,162 and three total lead-based paint decontamination costs ranged from €133.1 to €342 million, which were performed on the 37,382 houses concerned, in one shot for one year.

### Net benefits of the removal of lead-based paint in the French houses in 2008

We first estimated total net benefit induced by the risk factors soils and dust which contributed relatively more to low B-Pb values than to high B-Pb levels. This net benefit would stem from the reduction of lead hazard exposure and of childhood lead poisoning cases induced by this factor in respect of the costs C*_paint _associated with the control of lead environmental pollution. According to the hypothetical threshold values, they ranged from € 3.78 billion, € 1.88 billion and €0.25 billion respectively for children aged 1-6 years in the 2008 cohort, as shown in Table 6.

**Table 6:** Net benefits of the removal of lead-based paint in French houses (in €_2008 _Billion)

Blood-lead concentrations range (μg/L)	Benefits	Abatement costs	Net benefits	Hypothetical threshold values (μg/L)	Net cumulated benefits
**15 ≤ B-Pb < 24**	1.92	0.016 (0.008-0.02)	**1.90 (1)**	**B-Pb ≥ 15**	**3.78 (1+2+3)**

**24 ≤ B-Pb < 100**	1.64	0.016 (0.008-0.02)	**1.63 (2)**	**B-Pb ≥ 24**	**1.88 (2+3)**

**B-Pb ≥ 100**	0.33	0.074 (0.037-0.104)	**0.25 (3)**	**B-Pb ≥ 100**	**0.25**

Table 6 presents the net benefits of the removal of lead-based paint in French houses. Lead in soils and dust from the lead-based paint in homes built before 1949 represented 16% and 74% of cases of childhood lead intoxication for B-Pb concentration 15-100 μg/L and for B-Pb concentration≥100 μg/L, respectively. We applied these percentages to calculate the total benefits and the total costs C*paint (with central estimates selected) of the removal of lead-based paint ranged from blood-lead concentrations. We obtained € 1.92 billion(=€11.99billion*16%) and €0.016 billion (=(€0.2453/w_30_)*16%)) for the 15-24 μg/L range, €1.64 billion (=€10.28*16%) and €0.016 billion (=(€0.2453/w_30_)*16%)) for the 24-100 μg/L range, and € 0.33 billion (=€ 0.44 billion*74%) and (=(€0.2453/w_30_)*74%)) for the ≥ 100 μg/L range, respectively. We thus calculated the net benefits of the removal of lead-based paint ranged from blood-lead concentrations: €1.90 billion (1), € 1.63 billion (2) and € 0.25 billion (3) for B-Pb concentration 15-24, 24-100 μg/L and B-Pb concentration≥100 μg/L, respectively. Based on the three hypothetical threshold values, above 15, 24 and 100 μg/L, we estimated also the total net benefit cumulated: €3.78 billions (1+2+3), €1.88 billion (2+3) and €0.25 billion (3), respectively.

Reported per child, and given the number of children across hypothetical threshold values (i.e number of children from ≥ 15 μg/L, from ≥ 24 μg/L and from ≥ 100 μg/L, respectively), the yearly estimate of net benefit per child (2008) ranged from €1,610, €2,710 and €46,878, respectively.

## Discussion

The aim of this paper was to provide an economic evaluation of the health impacts of children with lead exposure in France. Based on the assumption of the EFSA report [1], that there is no threshold of lead exposure, our study provides a range of annual benefits and partial costs estimated in order to highlight the economic impact for society of lead exposure reduction policies below the conventional B-Pb screening value of 100 μg/L. Several hypothetical threshold values for intoxication (15, 24, 100 μg/L, respectively) were chosen following a "what if" approach. We have no strong data to choose levels lower than 15 μg/L but also do not assume it to be a safe exposure level. The partial cost benefit analysis documents a clear cost effectiveness of lead hazard control, which should result in benefits greatly superior to the costs, as suggested by the comparison of the sum of benefits to that of congruent costs for one year. This study showed that by reducing childhood lead exposure, large social benefits might be produced for the birth cohort of 2008 (and subsequent years): € 22.72 billion, € 10.72 billion and € 0.44 billion, respectively. The benefits were mainly due to the social avoided costs, specifically the lost life time earnings, at exposures corresponding to B-Pb <100 μg/L. There are some limitations to our analysis, due in particular to access to figures related to avoided costs and to costs of exposure reduction as we will see below. This is the reason why we could not perform a complete CBA. Direct health costs were also estimated but they were probably underestimated. Lead exposure provokes other health impacts besides cognitive disorders which were not assessed in this paper, such as cardiovascular diseases and cancer that lead to premature mortality. This would yield higher social costs than IQ decrement alone [56]. We disregarded for instance, drug costs and medical intervention costs such as intravenous chelation. Among other costs, the pretium doloris calculated on the basis of €8,000 per child in the Metal Blanc judgment was certainly underestimated, because only a small part of the children have been compensated, while also neglecting the psychological and economic suffering of the family or household of the children affected. We also estimated the need for special education to be 10% for children with B-Pb ≥ 100 μg/L. The somewhat uncertain data on crime costs suggest that the economic impact is comparatively low, but the costs of crime and rape were probably underestimated, because they did not include the value of statistical life, which may be greater than that of accidents (between €_1999 _0.5 to 1.5 million in Europe and French estimations were the lowest bracket estimate) [57,58].

They highlight the additional social consequences of lead pollution. In regard to annual costs to invest in pollution abatement, our preliminary estimates are affected by the paucity of available data. We could not make a complete CBA because of lack of available data on the abatement costs, we had a very small part of the industrial costs. Official data from the ministry of Environment show that the major industrial sources of lead in France are the metals and non metallic minerals sectors [59]. Three quarters of the 2007 emissions took place through water, and two waste treatment facilities alone amounted to 60% of total emissions of the ten most emitting facilities [60]. We had also quite imprecise cost estimates for substitution of lead pipes, whose yearly estimates are certainly exaggerated. So far, clean-up costs of industrial lead-contaminated sites cannot be evaluated in France. Partial data stem from the experience of the highly polluted MetalEurop site remediated by SITA-Suez Environment, which amounted to €28 million [61]. Unfortunately, these findings cannot be extrapolated to the national situation. As to contaminated sites, we point out the need for a specific evaluation. However, costs to decontaminate French houses with lead-based paint were available. And we calculated these costs once-for-all in one year, even if we overestimated the annual expenses, they appeared to be the most important efforts to be made in order to control the hazard. We could express an equivalent annual cost by using the capital recovery factor of standard interest calculations for loans which is the appropriate conversion factor. However, uncertainties remain regarding the time horizon and the social discount rate to use. A 0.05 conversion factor between one-time cost and annual cost is a compromise.

Some of the costs were paid within one year or paid over no more than five years, costs would be substantially less subsequent to that, while benefits would continue to accrue for each new birth cohort being born during the following years.

Our first estimates of total net benefit induced by reducing exposure to soils and dust in respect of the costs incurred by the decontamination of French houses with lead-based paint highlight that policies aimed at reducing lead exposures had an overall positive societal and economic impact. Additional estimates of total net benefit were performed, that considered the costs associated with dust and soils and drinking water lead pipes substitution. The expected health gains, according to the different B-Pb hypothetical threshold values, were calculated to be € 3.9 to 4 billion, € 1.86-2 billion and €0.12-0.25 billion respectively. The corresponding figures per child range from €1,661 to €1,721, €2,666 to €2,861, and €21,939 to €47,815, respectively. These estimates should be considered with caution, because of the uncertainty in the quality of data on costs of lead water pipes removal; a specific evaluation is also needed here.

Various uncertainties exist in our calculations: benefits linked to the dose-response function, and monetary valuation of the abatement costs linked to houses remediation, which yield uncertainties in the partial cost benefit estimates. According to Rabl and colleagues, there is a factor two uncertainty, both in the dose-response function and in the monetary valuation [62,63]. Should the scientific literature show some day evidence of lower toxicity level values than the one we used in this sensitivity analysis, the health cost figures would be substantially increased.

The overall return of investments is important and must be taken into account by the policy makers. They are in line with several US findings that illustrate how reduction of childhood lead exposure has a high social benefit, in particular the studies from Schwartz [16], Salkever [17] and Grosse and colleagues [64]. Between 1976 and 1999, Grosse et al. [64] estimated the economic impact of the trend of reduced lead exposure over 25 years in a cohort of children starting at 2 years of age in 2000. The estimate cost was valued from $110 to $319 billion (US) for the cohort each year, comparing it as if the blood lead concentration were that same as in 1975. Landrigan et al. [34] estimated the total annual costs of childhood lead poisoning to be $_1997_43 billion in each birth cohort exposed to lead in the US. Their methodological approach was based on the contribution of environmental pollutants by using an Environmentally Attributable Fraction (EAF) model, which was estimated at 100% for lead poisoning. Recent studies calculated the economic impact of childhood poisoning below100 μg/L. The most recent major U.S. study was that of Gould [8]. It was more comprehensive than those previously published, and produced a CBA by comparing the estimated costs in 1996 related to cleanup of lead-containing paint in the U.S. ($ 1 - $11 billion (US)) and secondly, by calculating the monetary benefits and social benefits by reducing lead exposure for a cohort of children <6 years ($192 - $270 billion) with earning losses amounted to 87% of total avoided costs. Total net benefits amounted to $ 181 - $ 269 billion. Therefore, a specific calculation induced by lead-based paint was not performed in this study. Muennig et al. [65], whereas, provided information on the benefits that might be realized if all children in the United States had a blood lead level of less than 10 μg/L. The net societal benefits showed improvements in high school graduation rates and reductions in crime would amount to $50,000 (SD, $14,000) per child annually at a discount rate of 3%. This would result in overall savings of approximately $1.2 trillion (SD, $341 billion) and produce an additional 4.8 million QALYs (SD, 2 million QALYs) for the US society as a whole.

Researchers in other European countries with prevalence of lead exposures similar to French figures may use this as a guide as to undertake similar economic assessments. Additionally, these data may motivate the revision of the current French policies as to whether or not to intervene in regard to lead pollution, and, in a more general sense, revamping France's overall policy on reducing pollution that may be affecting children's development. The introduction of unleaded petrol has greatly decreased emissions of lead in the atmosphere in France and globally. (Paris ambient air concentrations decreased by 97% between 1991 and 2005)[66]. The relative benefits of this action were substantial [3] and likely much greater than the benefits from further reduction of B-Pb levels today. Nonetheless, much abatement remains to be done, as other sources are only slowly being removed, if at all. The screening of houses for sale or rent with lead-based paint was implemented through the 2004 Public Health Act and its stringent policies on industrial emissions were triggered by EU regulations. The French 2004 national environmental health action plan has also contributed to the steady decrease in exposure of the general population and of its most vulnerable young segment over the last years in France.

EFSA recommends that "work should continue to reduce exposure to lead, from both dietary and non-dietary sources" [1]. The major prevention campaigns aim to reduce lead exposure to the lowest possible level in order to protect children and childbearing age women. The obtained benefits for exposure levels <100 μg/L in this study are in line with the EFSA recommendations. They are a first step evaluation which should be expanded and refined. Our results emphasize the substantial monetary advantages obtained from preventing losses of a few IQ points because of lead exposures among children. While 1-point change in Full Scale IQ score is within the standard error of an individual's single measurement, it may be highly significant on a population basis [25].

## Conclusions

The primary economic benefits of policies focused on lead exposure abatement are the further reduction of low blood lead levels. In contrast, prevention of cases with B-Pb >100 μg/L accounts for much lower benefits. This is because children with milder exposures are much more common and they still benefit from decreased exposure, as there is no known safe level of lead exposure. Lead toxicity is still a serious public health issue, despite the present low prevalence of unacceptably high B-Pb concentrations. Public policies to prevent lead exposure will reduce future medical expenses and the reduce the burden on special education classes. More importantly, they will also increase the productivity of children during their adult lives. Our CBA results suggest that overall reduction of costs due to toxicity can be achieved by further control of major contact media, including food, through diffusion of lead in the environment from industrial releases and also by further control of residential sources (leaded paint, deteriorated housing, old water pipes). In addition to abating the burden of developmental impairment in general, these policies will also help to reduce health disparities. This objective calls for prioritized policies focused on the most highly exposed communities and individuals. This combined strategy is a policy issue that our data aim to inspire. Yet, additional documentation of the B-Pb values for further evaluation is needed. A more thorough evaluation of the marginal costs of the measures to be taken is also needed in order to balance lead exposure abatement options.

## List of Abbreviations

AFSSA: French Food Safety Agency; ANAH: National Agency of the Housing Environment; B-Pb: blood-lead; CBA: cost benefit analysis; CEPA: California Environmental Protection Agency; COI: Cost of illness; CSHPF: High Council of Hygiene; EAF: Environmentally Attributable Fraction; EFSA: European Food Authority Safety; GDP: Gross Domestic Product; IEGRE: European Institute Reasoned Management for the Environment; IME: Medical Educational Institutes; INERIS: National Institute for Industrial Environment and Risks; INSERM: National Institute of Health and Medical Research; INSEE: National Institute for Statistics and Studies; InVS: French Institute for Public Health Surveillance; IQ: Intellectual Quotient; ITEP: Educational and Therapeutic Institutes; LERES: Laboratory study and research in environment and health; PPP: Purchasing Power Parity; QALY: Quality-Adjusted Life Year; SNSPE: French National system of surveillance of children's B-Pb concentrations.

## Competing interests

The authors declare that they have no competing interests. PGr is an editor-in-chief of Environmental Health, but was not involved in the editorial handling of this manuscript.

## Authors' contributions

CP performed the literature review, drafted the manuscript and carried out the analysis. MB, DZN, PGr, PGl and PH contributed substantially to defining the methods of the analysis, interpreting the results of the study and editing the manuscript. All authors read and approved the final version.
